# Klotho Function in Neurogenesis and Oligodendrogenesis: A Mini-Review Update

**DOI:** 10.1007/s12031-026-02487-z

**Published:** 2026-02-24

**Authors:** Isabela Ribeiro Possebom, Geovana Rosa Oliveira dos Santos, Elisa Mitiko Kawamoto

**Affiliations:** Department of Pharmacology, Institute of Biomedical Sciences, University of São Paulo, São Paulo, 05508-900 SP Brazil

**Keywords:** Klotho, Neurogenesis, Embryonic neurogenesis, Adult hippocampal neurogenesis, Oligodendrogenesis, Aging-related disease

## Abstract

Klotho is an anti-aging protein with multiple functions in maintaining homeostasis within the central nervous system (CNS). Klotho-induced neurogenesis and oligodendrogenesis play a critical role in myelination, neuroplasticity, and hippocampus-dependent cognition. In this context, the objective of this review is to synthesize well-established information about the mechanisms through which Klotho influences neurogenesis and oligodendrogenesis. Previous studies have demonstrated that Klotho regulates adult neurogenesis, stimulates the proliferation of neural progenitor cells (NPCs), promotes the differentiation of NPCs into neurons, and facilitates neuronal maturation and dendritic arborization. Additionally, Klotho stimulates oligodendrocyte differentiation and maturation, as well as myelination throughout life. The reviewed evidence suggests that Klotho influences cell fate decisions during both development and adulthood and has a modulatory role in neuronal fate determination from embryonic stem cells in neurogenesis and oligodendrogenesis. Therefore, Klotho may represent a promising therapeutic target for neuroprotection, including the prevention of neurodevelopmental and neurodegenerative diseases.

## Introduction

Klotho is known as an anti-aging protein; its nomenclature derives from Clotho, a figure in Greek mythology responsible for spinning the thread of life (Boksha et al. 2017; Dubal et al. 2014). Klotho protein was first described by Kuro-o and collaborators in 1997, which demonstrated that a mutation in both alleles of the *KL* gene significantly reduced Klotho expression, resulting in a characteristic phenotype of premature aging (a life expectancy of approximately 8 weeks) (Kuro-o et al. 1997). This accelerated-aging phenotype is marked by age-related disorders, including cognitive and motor dysfunction, emphysema, osteoporosis, atherosclerosis, gait disturbance, and infertility (Boksha et al. 2017; Kuro-o et al. 1997; Salech et al. 2019; Laszczyk et al. 2017). In contrast, Klotho-overexpression mice have a life expectancy extension of over 20% and improved learning and memory. Interestingly, KL-VS polymorphism in the human *klotho* gene is associated with increased life expectancy and enhanced cognition in heterozygous carriers (Boksha et al. 2017; Dubal et al. 2014; Salech et al. 2019).

Klotho protein has two homologous forms, α-Klotho and β-Klotho, which have been identified in both rodents and humans. Although the homologous forms exhibit 41% gene similarity, they have distinct functions, display different patterns of tissue expression, and act as co-receptors for different receptors (Boksha et al. 2017). α-Klotho protein is encoded by the *KL* gene, which produces two mRNAs that generate transmembrane and soluble Klotho. The transmembrane form of Klotho comprises two extracellular domains (KL1 and KL2), which are cleaved by ADAM10 and ADAM17, resulting in the release of soluble Klotho into the blood and cerebrospinal fluid (Boksha et al. 2017; Dubal et al. 2014; Kuro-o et al. 1997). Moreover, α-Klotho is highly expressed in the kidney and brain. In the central nervous system (CNS), α-Klotho is most abundant in the choroid plexus and expressed at lower levels in other areas such as the cortex, hippocampus, and cerebellum (Boksha et al. 2017; Dubal et al. 2014; Salech et al. 2019). Conversely, β-Klotho is encoded by the KLB gene and is highly expressed in the pancreas, intestine, and adipose tissue (Kuro-o 2019). Up to this point, there is no evidence for the existence of secreted β-Klotho forms (Boksha et al. 2017).

Klotho protein homologs are important endocrine components, since they are necessary for the high-affinity binding of fibroblast growth factor 19, 21, and 23 (FGF19, FGF21, FGF23) to their respective FGF receptors (FGFRs) that modulate several aspects of metabolism. The FGF23 is secreted by bone in response to phosphate intake and binds to α-Klotho-FGFR complexes on the kidney to regulate mineral excretion and homeostasis (Kuro-o 2019; Kurosu et al. 2005). Furthermore, FGF21 is a liver-derived factor that modulates responses to fasting and stress by stimulating lipolysis in adipocytes and activating the hypothalamus-pituitary-adrenal axis. Additionally, FGF19 is a bile acid response hormone secreted by the intestine that promotes metabolic responses to feeding in hepatocytes (Kuro-o 2019; Ogawa et al. 2007). In this context, α-Klotho maintains the phosphate/calcium/vitamin D homeostasis via FGF23-mediated signaling. At the same time, β-Klotho is a cofactor that increases the affinity of FGF21 and FGF15 for their respective receptors (FGFR1c and FGFR4), consequently activating a signaling cascade involved in glucose metabolism and fatty acid biosynthesis (Boksha et al. 2017; Kuro-o 2019; Kurosu et al. 2005).

Klotho exhibits anti-aging, anti-inflammatory, and antioxidant properties in various physiological systems (Salech et al. 2019; Laszczyk et al. 2017). Given the physiological functions promoted by Klotho, human studies have demonstrated that disruptions in Klotho expression increase the risk for multiple age-related disorders, such as chronic kidney disease (CKD), arteriosclerosis, diabetes, and obesity, as well as various types of cancer and neurodegenerative diseases (Kuro-o 2019; Clinton et al. 2013). It was reported that patients with comorbidities, such as type 2 diabetes, obesity, and cardiovascular diseases, have a decreased level of FGF21. Moreover, FGF21 may play a role in modulating the FGF-Klotho complex and influencing the aging process, as evidenced by FGF21 overexpression extending lifespan in mice (Kuro-o 2019). Given that the β-Klotho/FGF21 axis regulates glucose metabolism, disruptions in this axis impair adipose tissue’s ability to promote glucose uptake and insulin sensitivity. Furthermore, reduced β-Klotho expression in pancreatic β-cells was associated with dysfunction in insulin storage and secretion, as well as elevated glucose levels. In this context, FGF21 and FGF19 analogs, as well as agonistic antibodies against β-Klotho or the FGFR1-β-Klotho complex, are promising strategies for treating diabetes and obesity (Hua et al. 2021; Jackson et al. 2015). In contrast, α-Klotho plays a role in mechanisms against pathogenic processes in neurodegenerative and neuropsychiatric diseases, exerting several neuroprotective actions by inhibiting pathways such as insulin growth factor (IGF-1)/phosphatidylinositol kinase/protein kinase B/mechanistic target of rapamycin complex 1 (IGF-1/PI3K/Akt/mTORC), Wnt signaling, transforming growth factor β (TGF-β), and nuclear factor κB (NF-κB), and interacts with the Na^+^, K^+^-ATPase pump (Salech et al. 2019; Kurosu et al. 2005; Ogawa et al. 2007; Zeldich et al. 2014). Also, α-Klotho exerts neuroprotective effects by modulating components of the glutamate neurotransmitter system and the antioxidative system, as well as regulating neurogenesis, oligodendrocyte differentiation, and myelination (Mytych 2022; Bond et al. 2015; Prokhorova et al. 2019). Therefore, α-Klotho protein may act as a therapeutic tool for diseases associated with pathological brain aging (Prokhorova et al. 2019).

Furthermore, a significant body of studies has demonstrated that α-Klotho promotes the proliferation and differentiation of progenitor cells across several physiological systems (Kawaguchi et al. 1999; Shimada et al. 2004; Chihara et al. 2006; Fan and Sun 2016; Du et al. 2022; Madathil et al. 2014; Naeeni et al. 2023), including osteoblasts and osteoclasts (Kawaguchi et al. 1999) and endothelial progenitor cells (Shimada et al. 2004). Klotho deficiency in mice impairs both osteoblast and osteoclast differentiation (Kawaguchi et al. 1999), as well as angiogenesis and vasculogenesis (Shimada et al. 2004). Moreover, Klotho-mutant mice (*Kl*^*−/−*^) present a decrease in the number of osteoblast and endothelial progenitor cells compared to Klotho-wild-type mice (*Kl*^*+/+*^) (Shimada et al. 2004). Furthermore, Klotho overexpression in a preadipocyte cell line (3T3-L1) promotes adipocyte differentiation (Chihara et al. 2006). In contrast, *Kl*^*−/−*^ mice show decreased proliferation and differentiation in adipose-derived stem cells. In this context, treatment with recombinant soluble Klotho reverses adipogenic differentiation damage (Fan and Sun 2016). However, deletion of the Klotho gene or a lower concentration of soluble Klotho increases erythropoiesis. It decreases the hematopoietic stem cell (HSC) pool size, since soluble Klotho secreted by the kidney directly maintains HSC pool size (Du et al. 2022; Madathil et al. 2014). In summary, it is well established that the Klotho protein plays a vital role in maintaining the pool of progenitor cells and in their differentiation into various tissues. However, the specific role of Klotho in CNS cellular proliferation and differentiation remains incompletely elucidated.

Particularly, a study involving an in vitro MSC-to-neuron transdifferentiation model showed that a higher expression of Klotho mRNA is associated with morphological changes in mesenchymal stem cells (MSCs) and an increased expression of neuron-specific genes, such as NeuN and neurofilaments (NfL) (Naeeni et al. 2023). Moreover, an enhanced Klotho expression in ischemic injury is associated with a neuroprotective effect. A treatment with adipose-derived MSCs reduced cerebral ischemia injury in rats by decreasing pro-inflammatory and apoptotic factors, which results in improving cognitive performance and increasing the α-Klotho and α subunit of AMP-activated protein kinase (AMPK) expression compared with ischemic rats without treatment (Ranjbaran et al. 2022). Therefore, Klotho may be associated with a neuroprotective effect after injury and with MSC differentiation into neurons.

**Fig. 1:**
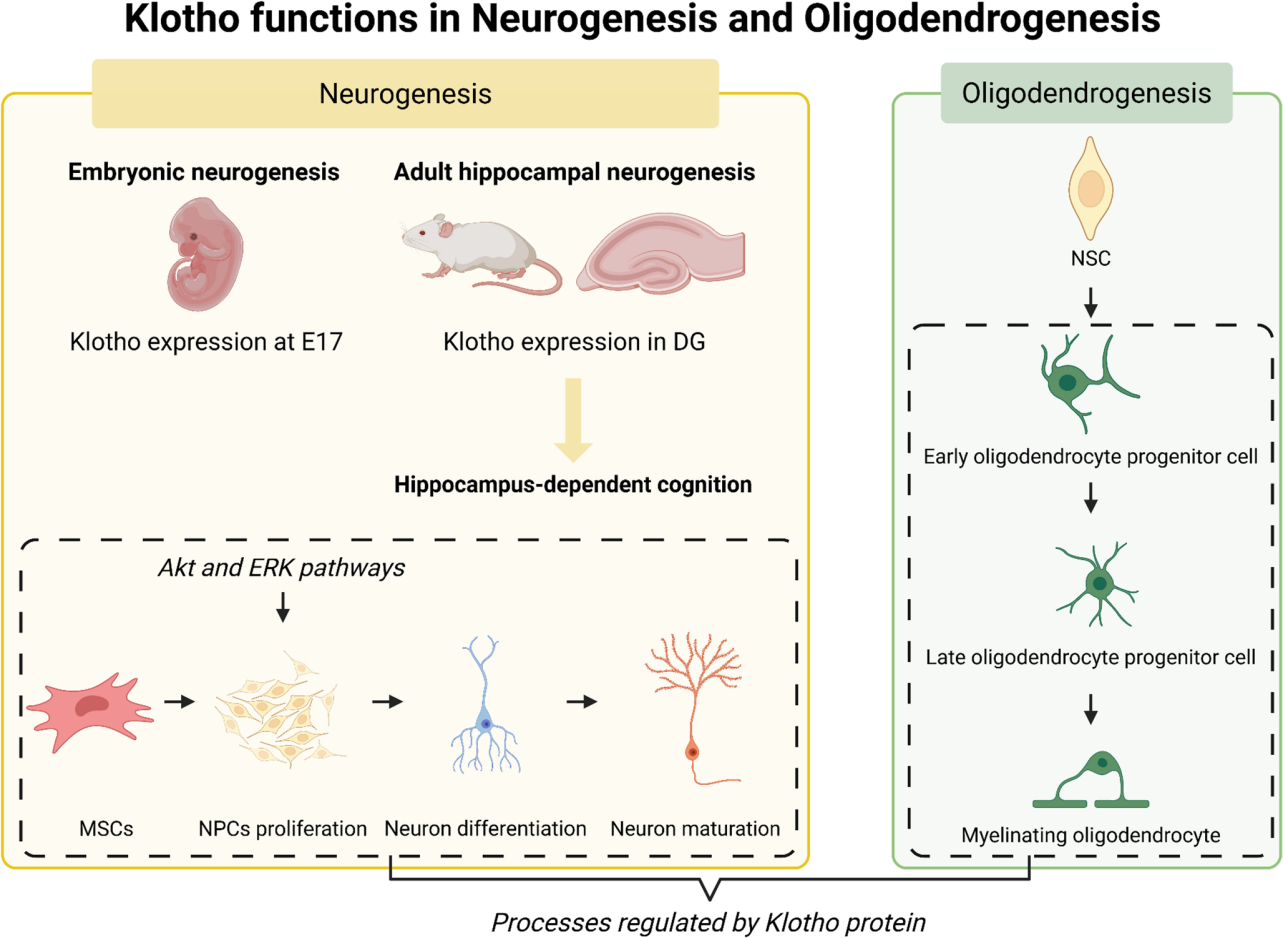
Klotho plays a role in neurogenesis and oligodendrogenesis throughout life. Klotho protein is expressed during development and throughout life. Klotho is involved in the differentiation of mesenchymal stem cells (MSCs) into neural progenitor cells (NPCs) and their subsequent proliferation, neuron differentiation, and maturation. Klotho also modulates oligodendrocyte differentiation and maturation, as well as the myelination process. DG: dentate gyrus; NSC: neural stem cell. Created in BioRender (https://BioRender.com/8zll1lm)

Furthermore, a significant set of studies has demonstrated that α-Klotho plays an important modulatory role in neurogenesis (Dubal et al. 2014; Salech et al. 2019; Laszczyk et al. 2017; Mytych 2022) and oligodendrogenesis (Chen et al. 2013, 2015), which occurs from development to adult life (Dubal et al. 2014; Salech et al. 2019; Laszczyk et al. 2017; Clinton et al. 2013; Chen et al. 2013, 2015; Kim et al. 2025). Neurogenesis is the process of generating new adult neurons from neural progenitor cells (NPCs), and oligodendrogenesis is the process of generating oligodendrocytes from parenchymal oligodendrocyte precursor cells (OPCs) and progenitors from the subventricular zone (SVZ) (Waly et al. 2014; Villalba et al. 2021). In this context, higher levels of Klotho expression promote cell fate decisions in neurogenesis and oligodendrogenesis during the embryonic phase, which are essential for the formation of neural circuits and myelination (Chen et al. 2013, 2015; Kim et al. 2025; Villalba et al. 2021). Klotho also improves hippocampal neurogenesis (Dubal et al. 2014; Salech et al. 2019) and oligodendrocyte differentiation and functionality throughout life (Chen et al. 2013, 2015). However, little is known about the specific functions by which Klotho modulates neurogenesis and oligodendrogenesis across the different stages of these processes and throughout various phases of development. This review aims to synthesize well-established information on the role of Klotho in neurogenesis, oligodendrogenesis, and its related mechanisms. Figure 1 synthesizes neurogenesis and oligodendrogenesis processes regulated by the Klotho protein.

## Klotho Function in Neurogenesis

Neurogenesis is the process of generating new neurons through an orchestrated series of transcriptional and morphological modifications from neural progenitor cells (NPCs) (Bond et al. 2015; Silva Siqueira et al. 2021). Neurogenesis occurs from the development until adult life, including several phases: proliferation, migration, differentiation, and circuit integration of mature neurons (LiCausi and Hartman 2018; Araki et al. 2021). Moreover, Klotho-induced neurogenesis in the postnatal phase enhances neuroplasticity and hippocampal-dependent cognition in mice and rats (Dubal et al. 2014; Salech et al. 2019; Laszczyk et al. 2017; Mytych 2022). Furthermore, dysregulation of embryonic neurogenesis can lead to malformations and functional disturbances characteristic of neurodevelopmental disorders. Therefore, the proper progression of embryonic neurogenesis supports healthy neurodevelopment (Villalba et al. 2021). In this context, a recent study has demonstrated that Klotho promotes embryonic neurogenesis. Moreover, Klotho-induced neurogenesis in the postnatal phase enhances neuroplasticity and hippocampal-dependent cognition in mice and rats (Dubal et al. 2014; Salech et al. 2019; Laszczyk et al. 2017; Mytych 2022). Therefore, Neurogenesis plays an important role in CNS homeostasis, highlighting its potential as a therapeutic target for neurodegenerative and neuropsychiatric disorders (Prokhorova et al. 2019; Vo et al. 2018). Table 1 summarizes the main findings about the role of Klotho in neurogenesis in the literature.

**Table 1:** Summary of the main results found in studies of klotho’s role in neurogenesis

	Klotho expression alterations	Main observations
Embryonic neurogenesis		
Kim et al., 2025	α-Klotho-knockdown in embryonic NPCs	- Reduced proliferation rate, decreased NPCs diameter, and decreased Ki67 labeling; - Repressed signaling pathways involving Akt and ERK phosphorylation; - Impaired differentiation of NPCs into neurons or oligodendrocytes; - Decreased survival of neural stem cells.
Kim et al., 2025	α-Klotho-overexpression in embryonic NPCs	- Enhanced proliferation rate, increased number of NPCs, and increased Ki67 labeling; - Enhanced differentiation of NPCs into neurons and oligodendrocytes; - Increased number of neurons with highly dendritic arborization.
Adult hippocampal neurogenesis		
Clinton et al., 2013	Quantification of Klotho mRNA expression	Detected Klotho expression in many brain structures on the first postnatal day.
Salech et al., 2019 Laszczyk et al., 2017 Vo et al., 2018 Shiozaki et al., 2008	*KL* knockout in mice	- Increased the adult hippocampal neurogenesis; -Decreased number and size of neurospheres, as well as the number of immature neurons, delayed neuron maturation, and impaired neuronal dendritic arborization; -Premature biochemical and morphological alterations, as well as reduced brain and spinal cord volume.
Salech et al., 2019	Local-hippocampus *KL* knockout mice	Impaired AHN, leading to a decrease in hippocampus-dependent cognition.
Laszczyk et al., 2017 Vo et al., 2018 Roig-Soriano et al., 2025	Recombinant Klotho supplementation	- Reverse the reduction in the NPCs proliferation rate in KL knockout mice; - Preventing premature aging of the NPCs niche, and spatial discrimination impairment in KL knockout mice; - Increased number of immature neurons.
Dubal et al., 2014 Laszczyk et al., 2017 Vo et al., 2018	*KL* overexpression in mice	- Enhanced expression of the immature neuron glutamate receptor subunit, GluN2B; - Increased NPCs’ proliferation rate, the number of immature neurons, and enhances neuronal dendritic arborization.
Dias et al., 2021 Setel et al., 2022 Su et al., 2019	Klotho upregulation	- Improved cognitive performance; - Increased number of neuroblasts, and increased neurogenic markers; - Klotho modulates the proliferation rate of the NPC pool.
Ho et al., 2020	Klotho downregulation	Decreased synaptic plasticity and neural progenitor cells in the hippocampus.
Laszczyk et al., 2019	FGF-23 receptor-deficient mice	Decreased neuronal differentiation.

### Klotho and Embryonic Neurogenesis and Oligodendrogenesis

In the developing brain, radial glia from the ventricular zone (VZ) divide into intermediate progenitors, which amplify and migrate out of the VZ to establish at the SVZ, where these progenitors divide and generate neuroblasts. Thus, the neuroblasts migrate through the cortex. In embryonic neurogenesis, neural stem cells (NSCs) divide to generate differentiated progenitor cells and maintain the stem pool (Engler et al. 2018).

In 2017, Laszczyk’s study group investigated the expression of Klotho during development using qPCR on the mRNA of mouse NPCs, detecting its first expression at 17 embryonic days (Laszczyk et al. 2017). Also, it was demonstrated that Klotho deficiency did not impair embryonic neurogenesis. In this study, only at 7 weeks postnatally, Klotho-deficient mice showed a decrease in the number of NPCs and immature neurons compared to wild-type mice. In addition, the study showed that hippocampal volume at 3 and 7 weeks of age was not significantly different between WT and KO mice. However, at 7-week-old KO mice exhibited a decrease in fimbria volume (Laszczyk et al. 2017). Furthermore, α-Klotho mutant mice show premature biochemical and morphological changes in synaptic proteins and terminals, microtubules, and organelles, as well as reduced brain and spinal cord volume. It is possible that this reduction in brain volume was caused by accelerated degeneration or developmental defects (Shiozaki et al. 2008).

Nevertheless, a recent study by Kim et al. demonstrated that α-Klotho may also play a role in embryonic neurogenesis and oligodendrogenesis. This study showed that α-Klotho is expressed in NPCs since the 13.5-day embryonic mouse. In addition, Klotho knockdown in embryonic NPCs reduced the proliferation rate, decreased the neurosphere diameter, and decreased the number of NPCs labeled with Ki67 (a proliferation marker). Furthermore, knockdown of α-Klotho repressed signaling pathways involving Akt and ERK phosphorylation, suggesting that this signaling may represent a possible mechanism by which Klotho promotes the proliferation of mouse embryonic NPCs (Kim et al. 2025). Moreover, knocking down α-Klotho also impaired the differentiation of NPCs into neurons or oligodendrocytes, but not astrocytes. In addition, a terminal deoxynucleotidyl transferase dUTP nick end labeling (TUNEL) assay showed that Klotho deletion increased basal cell apoptosis. This suggests that α-Klotho is an important factor in the survival of neural stem cells (Kim et al. 2025).

In contrast, overexpressing α-Klotho in transmembrane or secreted form in embryonic cells enhances the proliferation of NPCs (number of neurospheres and Ki67 labeling). Furthermore, Klotho overexpression also promotes neuron and oligodendrocyte differentiation, resulting in an increased number of neurons with highly dendritic arborization. In conclusion, Kim’s study indicates that α-Klotho promotes cell survival, NPC proliferation, and neuronal and oligodendrocyte differentiation (Kim et al. 2025). Therefore, Klotho also may play a modulatory role in neuronal fate determination from embryonic stem cells in neurogenesis and oligodendrogenesis.

### Klotho and Adult Hippocampal Neurogenesis

**Fig. 2:**
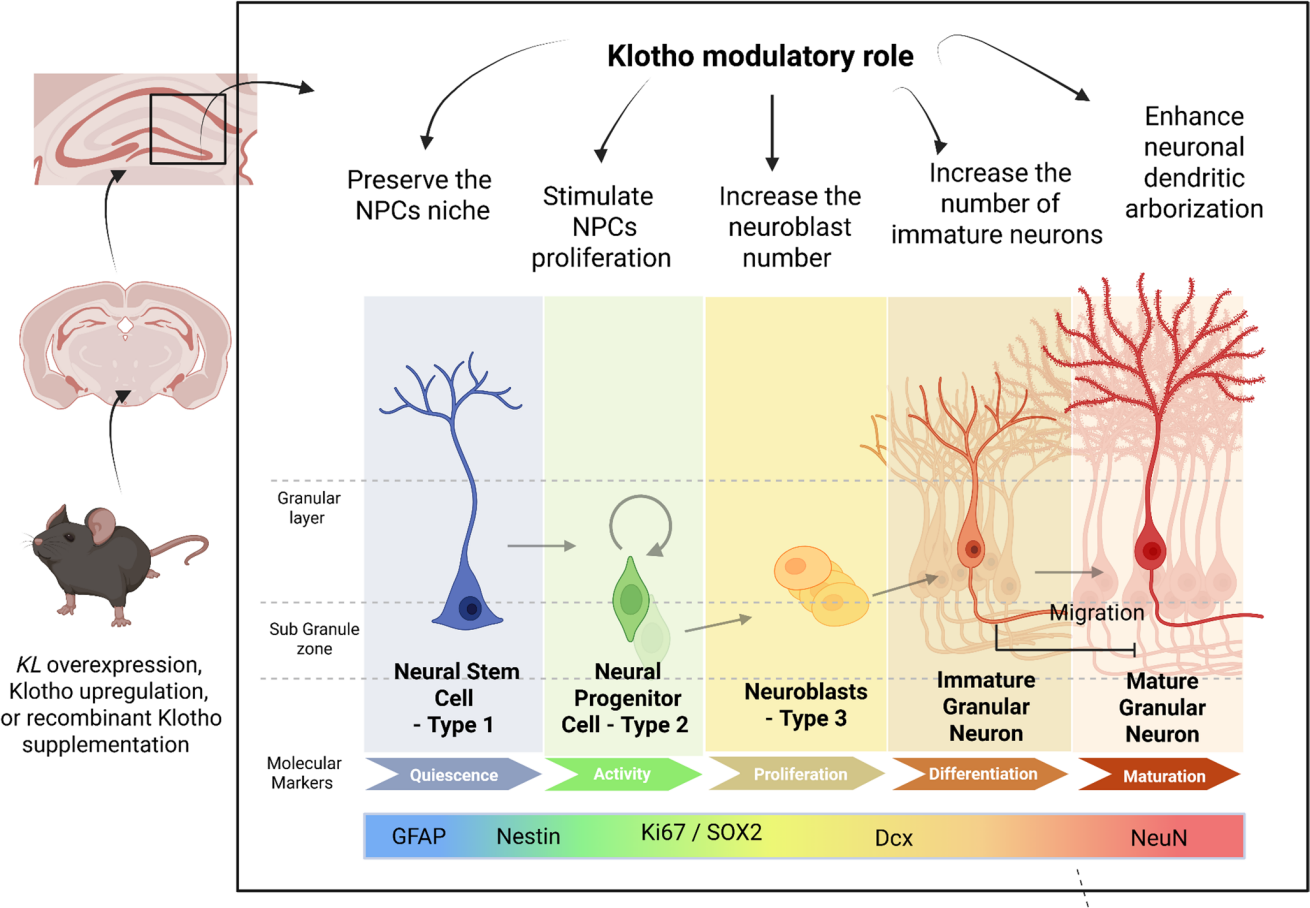
Klotho modulates distinct phases of hippocampal neurogenesis. The figure illustrates the stages of neurogenesis: proliferation, neuronal differentiation, and subsequent neuronal maturation

The adult hippocampal neurogenesis (AHN) evolves the neurogenic niche, which is localized in the SVZ of the lateral ventricles and in the subgranular zone (SGZ) of the hippocampal dentate gyrus (DG). The niche comprises cellular and non-cellular components that influence the fate of the NPC: self-renewal or differentiation (Kempermann et al. 2004; Li Ming and Song 2011). In this process, a bipotent radial glia-like stem cell (type-1 cell) has undifferentiated molecular markers such as nestin and glial fibrillary acidic protein (GFAP). Type 1 divides symmetrically for long-term expansion. However, type-1 cells can divide asymmetrically to give rise to lineage-committed progenitor cells (type-2 and type-3 cells), which subsequently differentiate into immature neurons or astrocytes. Some type 2 cells and all type 3 cells express doublecortin (DCX), a marker for immature neurons, while intermediate progenitors amplifying express SOX2 + and Ki-67. Ultimately, immature neurons differentiate into mature neurons as they migrate to the granule cell layer and begin to express NeuN, as shown in Fig. 2 (Salech et al. 2019; Laszczyk et al. 2017; Bond et al. 2015; Kempermann et al. 2004; Li Ming and Song 2011). The SGZ contains neural stem cell pools that generate new neurons throughout postnatal life, which are crucial for maintaining structural integrity, regenerating brain tissue, and promoting neuroplasticity (Isaev et al. 2019). This process ensures controlled cell expansion and neuronal formation, and prevents depletion of the NPC pool (Bonafina et al. 2020; Su et al. 2019).

In 2014, Cliton’s group investigated Klotho mRNA expression in postnatal developing and adult brains of rats with in situ hybridization. The analyses detected Klotho expression in many brain structures of gray matter as early as the first postnatal day. Additionally, it has been demonstrated that the *KL* gene is highly expressed in the DG, particularly in mature neurons (Clinton et al. 2013). Also, the literature has reported that Klotho overexpression may enhance hippocampus-dependent cognition by modulating adult neurogenesis, which probably influences the cellular decision of NPCs differentiation into neuroblasts through asymmetric division (Dubal et al. 2014; Salech et al. 2019; Laszczyk et al. 2017; Su et al. 2019).

Salech et al. showed that AHN is increased in *KL* knockout mice. In contrast, local downregulation of Klotho in the mouse hippocampus impairs the AHN process, leading to a decrease in hippocampus-dependent cognition. However, treatment with recombinant Klotho increases the proliferation rate of cultured NPCs and promotes their differentiation into neurons (Salech et al. 2019). The site-specific deletion of Klotho in the hippocampus generates distinct responses in the neurogenesis compared to whole-body deletion of the *KL* gene in mice. A possible hypothesis for this difference is that whole-body deletion may have generated compensatory responses that promote cell proliferation in this model, since second metabolites may cross the barrier. Furthermore, the hippocampus is a region composed of a neurogenic niche. Therefore, the local knockdown of Klotho may have a more direct influence on neurogenesis, impairing hippocampal-dependent memory (Salech et al. 2019).

Furthermore, posterior studies have reported that Klotho prevents spatial memory loss by promoting hippocampal neurogenesis in NPCs of postnatal mice (Dubal et al. 2014; Salech et al. 2019; Laszczyk et al. 2017; Vo et al. 2018). Laszczyk’s group has reported that *KL* gene deletion in hippocampal NPC decreased the number and size of neurospheres, as well as the number of immature neurons, delayed neuron maturation, and impaired neuronal dendritic arborization. Therefore, Klotho deficiency in mice impaired the proliferation process and compromised neuronal maturation, suggesting an early depletion of the neurogenic niche. In this context, supplementation with recombinant Klotho could reverse the decrease in NPCs proliferation, protect against premature aging of the niche, and mitigate spatial discrimination impairment. In contrast, Klotho overexpression increased the NPCs’ proliferation rate, the number of immature neurons, and enhanced neuronal dendritic arborization (Laszczyk et al. 2017; Vo et al. 2018). These results suggest that Klotho is essential to modulate different stages of adult neurogenesis.

Moreover, the results from Dubal’s group study in 2014 suggest that Klotho overexpression enhances the expression of the immature neuron glutamate receptor subunit, GluN2B. Elevated GluN2B protein levels were associated with neuroplasticity, since these receptors facilitate long-term potentiation (LTP) and improve cognitive ability in young mice. Therefore, since the N-Methyl-D-Aspartate (NMDA) receptor modulates the proliferation and differentiation of immature neurons, increasing GluN2B subunits could improve neurogenesis (Dubal et al. 2014).

Other studies have demonstrated an association between Klotho expression and the promotion of neurogenesis (Dias et al. 2021). Dias’ research group showed that female mice subjected to intermittent fasting exhibited an improvement in long-term memory retention, as well as a higher number of proliferating NPCs and neuroblasts, which were associated with an upregulation of the *KL* gene in the hippocampus (Dias et al. 2021). Furthermore, a similar association was explored in a model of caloric restriction and treatment with Astragalus, which is an antioxidant and anti-inflammatory herbal compound. In this study, increased neurogenic markers, such as doublecortin (DCX) and NeuN, and brain-derived neurotrophic factor (BDNF) levels, as well as improved cognitive performance, were associated with an enhancement of Klotho expression in the hippocampus (Setel et al. 2022). Similarly, a recent study has shown that treatment with recombinant soluble Klotho increases the number of immature neurons in a histological analysis of mice (Roig-Soriano et al. 2025). In contrast, Ho’s 2020 study shows decreased synaptic plasticity in the DG and CA1 regions of the hippocampus, as well as reduced neural progenitor cells in the DG, associated with decreased Klotho levels in C9orf72 knockout mice that simulate amyotrophic lateral sclerosis and frontotemporal dementia (Ho et al. 2020).

Since Klotho is a co-receptor of the FGF-23 receptor and performs part of its functions through receptor activation, FGF-23 deletion can also induce a hippocampal-dependent cognitive impairment in mice. However, FGF-23-deficient mice exhibited minor changes in hippocampal neurogenesis, characterized by decreased neuronal differentiation without significant differences in the proliferation phase (Laszczyk et al. 2019). These results suggest that FGF-23 deletion could not be related to neurogenesis impairment, whereas Klotho deficiency is associated with cognitive impairment due to an imbalance in neurogenesis. In conclusion, an increase in Klotho gene expression or circulating protein enhances pro-neurogenic parameters and their respective functional consequences. In contrast, lower levels of Klotho are associated with impaired adult neurogenesis.

In 2019, Su et al. demonstrated, using single-cell RNA sequencing, that α2-chimaerin (a Rho GTPase-activating protein) is essential for maintaining the NPC pool during adult neurogenesis. In this study, a subpopulation of NPCs with high Klotho expression in the hippocampus of α2-chimaerin knockout mice was shown to provide signals that modulate the proliferation rate of NPCs (Su et al. 2019). Moreover, decreased Klotho expression in the NPCs population is associated with premature cell differentiation and loss of the NPCs pool, which impairs synaptic plasticity and compromises hippocampal function (Dubal et al. 2014; Laszczyk et al. 2017; Vo et al. 2018; Su et al. 2019). These results corroborate previous studies demonstrating that Klotho deficiency decreases NPC proliferation and causes early depletion of the neurogenic niche associated with aging-related diseases in the CNS (Laszczyk et al. 2017; Vo et al. 2018; Su et al. 2019). Therefore, the data summarized in the diagram in Fig. 2 corroborate the hypothesis that the Klotho protein is important for cellular decision-making and the preservation of the NPCs pool during neurogenesis.

The scheme shows that higher levels of Klotho expression in KL-overexpression mutant mice, endogenous klotho upregulation, or recombinant Klotho supplementation promote neurogenesis. Increased klotho expression may preserve the NPCs’ niche, stimulate NPCs proliferation, increase the number of neuroblasts and immature neurons, and enhance dendritic arborization. The indiferentiated molecular markers are Nestin and glial fibrillary acidic protein (GFAP). The proliferation markers are SOX2 and KI67. Doublecortin (DCX) labels immature neurons, and NeuN labels mature neurons. Created in BioRender (https://BioRender.com/zn72n28).

## Klotho Function in Oligodendrogenesis

Oligodendrocyte precursor cells (OPCs) are a type of glial cell that persist in the CNS throughout the lifespan and differentiate into myelinating oligodendrocytes (Xiao and Czopka 2023). Myelination improves the conduction and provides metabolic support to the axons (Xiao and Czopka 2023). Moreover, the brain’s white matter tracts are formed by neuronal axons wrapped by myelin sheaths and oligodendrocytes that perform myelin maintenance (Xiao and Czopka 2023; Duce et al. 2008). Oligodendrogenesis and myelin formation are important processes for modulating neural circuits, learning, and memory (Hu et al. 2025). In early embryogenesis, neural stem cells (NSCs) from the ventricular zone generate OPCs, which migrate, proliferate, and differentiate into myelinating mature oligodendrocytes. Adult oligodendrogenesis is crucial for neuroplasticity and brain function, as oligodendrocytes are generated to myelinate previously unmyelinated axons or remodel existing myelin sheaths. Both oligodendrogenesis and the myelination process decline with increasing age, as they are associated with reduced OPC proliferation and differentiation (Hu et al. 2025). Table 2 summarizes the main findings about the role of Klotho in oligodendrogenesis in the literature.

**Table 2:** Summary of the main results found in studies of klotho’s role in oligodendrogenesis

	Klotho expression alterations	Main observations
Duce et al., 2008	Klotho downregulation	- Decreased volume and increased diffusivity in the forebrain white matter of rhesus monkeys.
Abe et al., 2016	Klotho upregulation	- Reverse a decline in oligodendrocytes in the white matter tissues of rats.
Chen et al., 2013	Recombinant Klotho supplementation	- Increased the number of primary oligodendrocytes, and enhanced the expression of myelin proteins in mice; - Downregulated 80% of genes in MO3.13 cells; - Stimulated ERK and Akt signaling pathways, which decrease the proliferative phases and increase differentiation of MO3.13 cells.
Chen et al., 2013	Klotho-deficient mice	- Reduced myelinated sheaths in the corpus callosum and optic nerve; - Decreased expression of myelin proteins and myelin-related genes; - Impaired organization of myelin sheaths and hypomyelination in individual axons.

### Klotho and Oligodendrocytes

The research of Duce et al. (2008) demonstrated that an age-related decrease of Klotho protein expression was correlated with volume loss and increased diffusivity in the forebrain white matter, suggesting that Klotho may play a role in disrupting white matter function during aging (Duce et al. 2008). Moreover, Klotho may perform a neuroprotective function in the brain’s white matter. In a model of neurotoxicity, rats subjected to maternal exposure to cuprizone had a significant decrease in the mature oligodendrocytes and myelination impairment. In this context, transcript upregulation of Klotho can reverse a decline in oligodendrocytes in the white matter tissues (Abe et al. 2016).

Klotho plays an important role in oligodendrocyte differentiation and maturation, and the development of the axon’s myelin sheaths. A treatment with recombinant Klotho increases the percentage of mature primary oligodendrocytes and enhances the expression of myelin proteins (Chen et al. 2013). In contrast, Klotho-deficient mice demonstrated a significant reduction in myelinated sheaths in the corpus callosum and optic nerve, as well as decreased expression of myelin proteins and myelin-related genes compared with control mice. Moreover, ultrastructural analysis showed that *KL*-deficient mice exhibit disorganized myelin sheaths and hypomyelination in individual axons (Chen et al. 2013). Additionally, an in vitro study of Klotho treatment in a human oligodendrocytic hybrid lineaMO3.13) had demonstrated that Klotho treatment showed widespread alterations in the expression of the differentially expressed0% of genes downregulated). Furthermore, Klotho stimulated ERK and Akt signaling pathways, which decrease the proliferative phases and increase differentiation of MO3.13 cells (Chen et al. 2015). These results suggest that Klotho is capable of broadly modulating the cellular decision to differentiate within the human oligodendrocyte lineage.

The data set showed that Klotho may have an important function in oligodendrocyte differentiation and the myelination process in different models. Klotho also plays a humoral factor role in maintaining the number of oligodendrocytes and axon myelin integrity throughout life. Therefore, since the quality of myelination is essential for the preservation of white matter tissue, Klotho function is possibly associated with the prevention of neurodegenerative diseases such as multiple sclerosis and schizophrenia (Chen et al. 2015; Xiao and Czopka 2023).

## Conclusion

Klotho regulates the differentiation of various progenitor cell populations, such as neural progenitors and oligodendrocyte precursors. Specifically in the CNS, Klotho plays a modulatory role in cell fate decisions of NPCs to proliferate or differentiate into neurons or oligodendrocytes during both embryonic and adult neurogenesis. Furthermore, Klotho maintains oligodendrocyte progenitor number, promotes oligodendrocyte differentiation and maturation, and oligodendrocyte-dependent myelin development and integrity. Moreover, the reviewed literature data suggest that a decrease in Klotho expression leads to an early depletion of the neurogenic niche and a failure in the neuronal differentiation process. In contrast, Klotho overexpression in a cell subpopulation reinforced the hypothesis that Klotho signals control the preservation of the NPCs pool. Furthermore, a significant set of studies has demonstrated that recombinant Klotho treatment or enhanced expression of this protein increases markers of neurogenesis and neuroplasticity, as well as improves cognitive performance. In conclusion, Klotho protein largely modulates the main processes of neurogenesis and oligodendrogenesis both in development and throughout life, and their respective functional consequences. These data are critical considering the potential impact of Klotho-induced neurogenesis on the correct development of neural networks, preservation of the NPCs pool, myelination, and cognitive function, as well as its potential contribution as a therapeutic tool for the prevention and treatment of neurodevelopmental and neurodegenerative diseases.

## Data Availability

No datasets were generated or analysed in the current study.
